# Case Report: Two cases of primary pulmonary endobronchial extranodal marginal zone lymphoma of mucosa-associated lymphoid tissue localized in the trachea and bronchus

**DOI:** 10.3389/fonc.2025.1626336

**Published:** 2025-08-14

**Authors:** Lei Sha, Meng-Yao Cai, Min Feng, Chang Dong

**Affiliations:** Department of Respiratory and Critical Care Medicine, First Affiliated Hospital of Dalian Medical University, Dalian, China

**Keywords:** primary pulmonary lymphoma (PPL), MALT lymphoma, wait and watch approach, PET-CT, transbronchial biopsy (TBB)

## Abstract

**Background:**

Primary pulmonary extranodal marginal zone lymphoma of mucosa-associated lymphoid tissue (MALT lymphoma) localized in the trachea and bronchus is rare and diagnostically challenging due to nonspecific clinical and radiological features.

**Case description:**

We present two cases of primary endobronchial MALT lymphoma incidentally diagnosed via bronchoscopy. We assessed the characteristic bronchoscopic finding of endobronchial MALT lymphoma, radiological examinations and overall treatment strategies.

**Conclusion:**

This study highlights the importance of considering endobronchial MALT lymphoma as a potential diagnosis. A thorough bronchoscopic evaluation of the central airways, along with appropriate biopsy of suspicious lesions, is crucial. Endobronchial MALT lymphoma should be suspected when targeting multiple widely stalked and smooth submucosal nodules or protrusions, mainly located in the central airways.

## 1 Introduction

**Figure 1:**
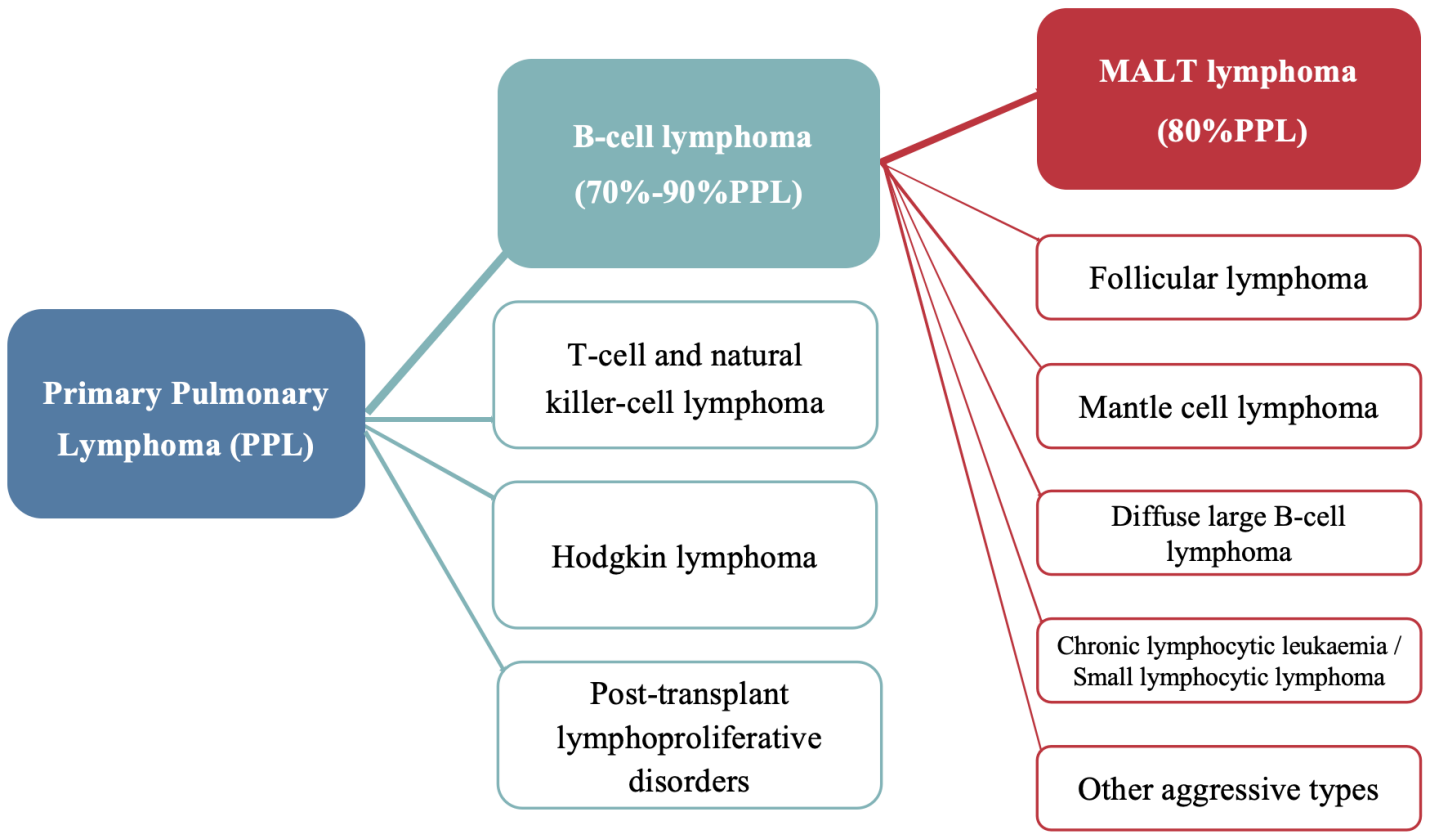
The proportion of pulmonary MALT lymphoma in primary pulmonary lymphoma.

Primary pulmonary lymphoma (PPL) refers to a clonal proliferation of lymphoid cells localized to the lung parenchyma and/or bronchi, with no evidence of extrapulmonary involvement at initial diagnosis or within the following three months (1). It accounts for only 0.5%–1% of primary pulmonary malignancies and <1% of non-Hodgkin’s lymphoma (2). Primary pulmonary extranodal marginal zone lymphoma of mucosa-associated lymphoid tissue (MALT) lymphoma is a type of primary pulmonary B-cell non-Hodgkin’s lymphoma, accounting for nearly 90% of such cases and 80% PPL (**Figure 1**) (3). However, primary endobronchial MALT lymphoma that is confined to the tracheobronchial tree remains very rare with only a few cases reported (4–6). Approximately half of patients with pulmonary MALT lymphoma are asymptomatic (7), and nonspecific computed tomography (CT) findings often lead to misdiagnosis, particularly in cases with isolated endobronchial involvement. Here, we report two cases of endobronchial MALT lymphoma incidentally diagnosed via bronchoscopy. The first case presented with pneumonia, and bronchoscopy examination revealed multiple nodular protrusions in the trachea and right middle lobar bronchus, with biopsy confirming MALT lymphoma. The second case presented with asymptomatic cavitary lung lesions, and MALT lymphoma with only tracheal involvement was diagnosed via transbronchial biopsy.

## 2 Diagnostic assessment

### 2.1 Case 1

**Figure 2:**
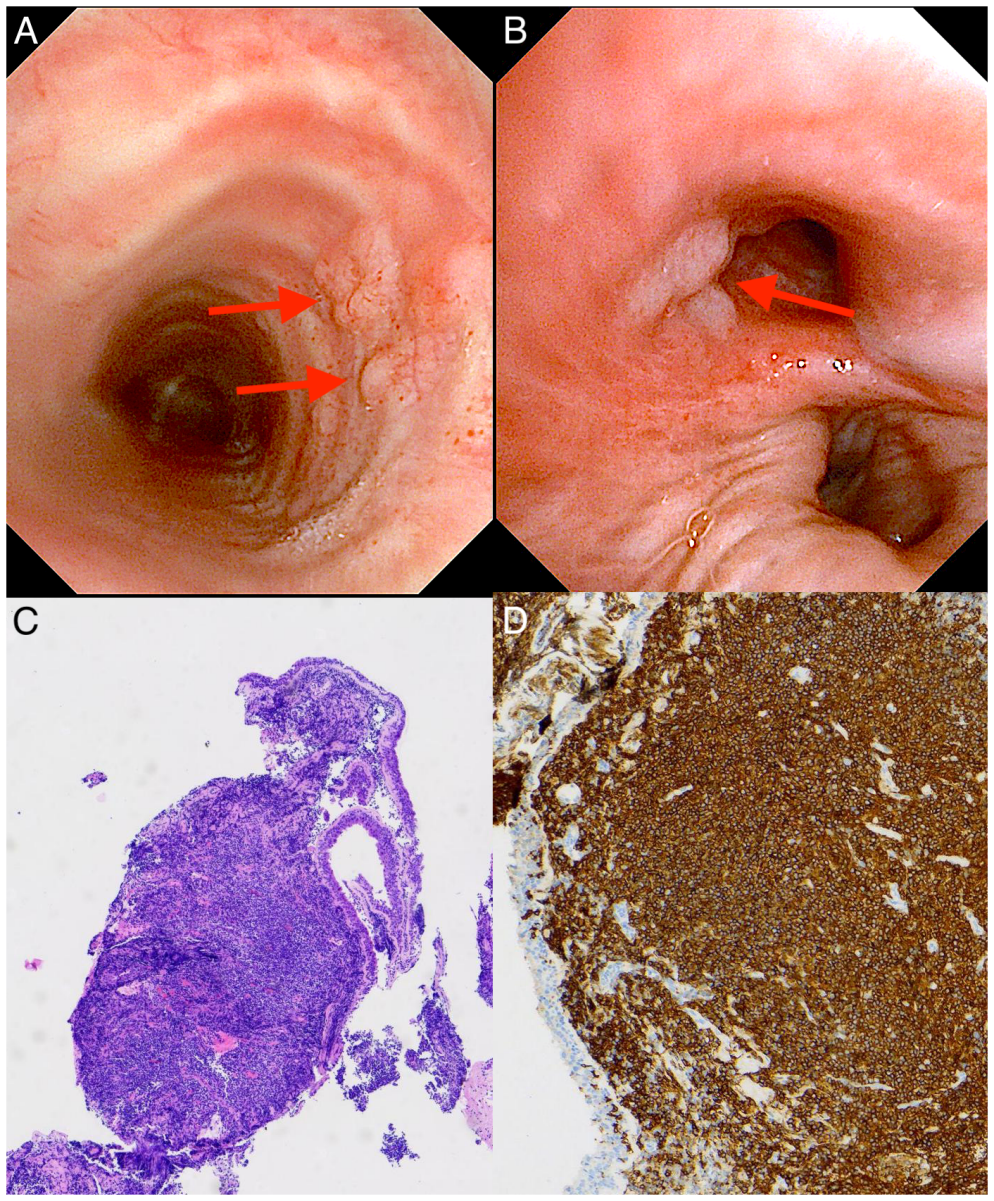
Bronchoscopic findings and pathological biopsy of case 1 with primary endobronchial MALT lymphoma. **(A, B)** Bronchoscopy showing multiple broad-based, smooth nodular lesions localized in the trachea [red arrows in **(A)**] and right middle lobar bronchus [red arrow in **(B)**]. **(C)** Light microscopic image showing diffuse diffuse infiltration of lymphoma cells, mostly centrocyte-like lymphocytes with deep blue nuclei. (Hematoxylin-Eosin stain; 40×magnification). **(D)** Immunohistochemical examination showing the cells were diffusely positive for B cell markers of CD20, manifested as dense brown staining of the cell membrane and pale blue staining of the nucleus (with hematoxylin counterstaining) (×100).

A 65-year-old man with diabetes and a history of smoking was admitted for a 10-day fever and 3-day dyspnea. The clinical information and laboratory results of the patient on admission were unremarkable and summarized in **Table 1**. Pulmonary function tests indicated that ventilation function was normal and diffusion function was slightly decreased. Chest CT revealed bilateral inflammatory infiltrates and mildly enlarged mediastinal lymph nodes. Bronchoscopy identified yellow-white secretions in the carina and bilateral main bronchus, and the testing of bronchoalveolar lavage fluid (BALF) for Mycobacterium tuberculosis, general bacteria and fungi smear, galactomannan tests and cryptococcus smear were all negative. Further BALF metagenomic next-generation sequencing revealed a positive result for Klebsiella aerogenes, with 100 uniquely mapped reads, which was consistent with the initial diagnosis of community acquired pneumonia. Unexpectedly, multiple broad-based, smooth nodular lesions were observed in the trachea and right middle lobar bronchus. Mucosal biopsy demonstrated a large number of lymphoid cells infiltration, and immunohistochemistry staining revealed CD20 (+), CD19 (+), Bcl-2 (+), Bcl-6 (partial+), CD21 (FDC net+), CD23 (FDC net+), CD3 (–), CD5 (-), CD10 (-), CD43 (-), Cyclin D1 (-), Ki67 (+20%) (**Figure 2**). The pathology was consisitent with MALT lymphoma. Positron emission tomography (PET)-CT showed mild 18 F-fluorodeoxyglucose (FDG) uptake with an average maximum standardized uptake value (SUVmax) 2.8-4.6 in hilar and mediastinal nodes.

**Table 1:** The clinical information of two pulmonary MALT lymphoma patients on admission.

Clinical information	Case 1	Case 2
Sex	male	female
Age, year	65	62
Chief compliant	fever and dyspnea	asymptomatic lung cavity
B symptoms	fever	none
Past medical history	diabetes	asthma, hypothyroidism
Smoking	yes	no
Cause of hospitalization	pneumonia	cavitary lung lesion
WBC, x10^9^/L	7.70	9.38
neutrophil, x10^9^/L	5.22	7.30
lymphocyte, x10^9^/L	1.71	1.52
PLT, x10^9^/L	272	153
Hb, g/L	150	141
ALT, U/L	19	17
AST, U/L	21	28
Creatinine, umol/L	65	67
CRP, mg/L	7.77	1.31
ESR, mm/h	25	17
PCT, ng/ml	0.23	0.21
CEA, ng/ml	1.22	1.31
TAP, um^2	119.459	103.342

MALT, Mucosa-associated Lymphoid Tissue; WBC, White Blood Cell Count; PLT, Platelet Count; Hb, Hemoglobin; ALT, Alanine Aminotransferase; AST, Aspartate Aminotransferase; CRP, C-reactive Protein; ESR, Erythrocyte Sedimentation Rate; PCT, Procalcitonin; CEA, Carcinoembryonic Antigen; TAP, Tumor Abnormal Protein.

### 2.2 Case 2

**Figure 3:**
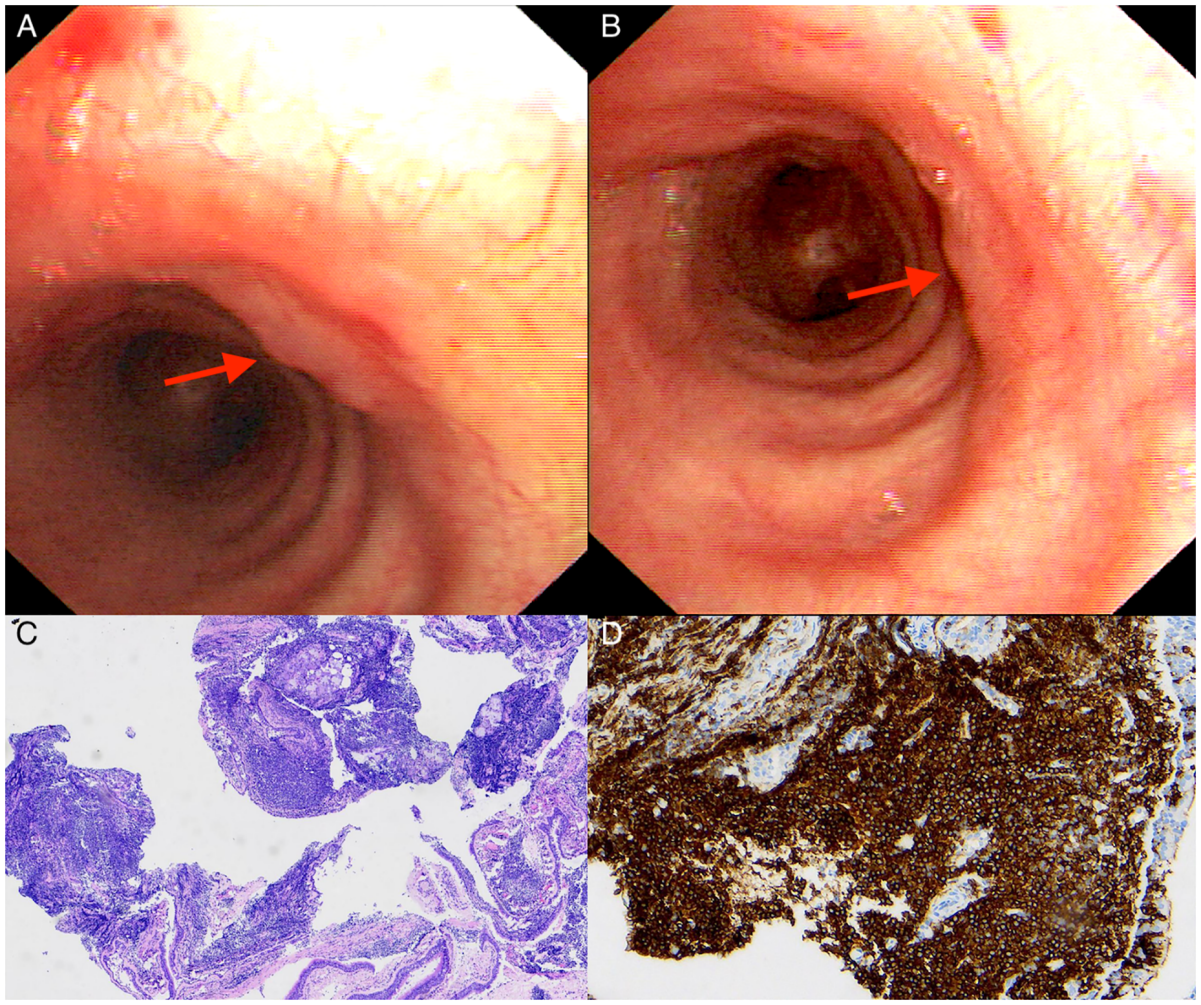
Bronchoscopic and pathological findings of case 2 with primary endobronchial MALT lymphoma. **(A, B)** Bronchoscopy showing several nodular protrusions covered with smooth bronchial mucosa along the trachea (red arrows). **(C)** Microscopically showing diffuse infiltration of lymphoma cells. (Hematoxylin-Eosin stain; 40×magnification). **(D)** The lymphoma cells were diffusely positive for CD20 (×100).

A 62-year-old asymptomatic woman was referred for a solitary cavitary lung lesion detected 18 months prior. Her clinical information and laboratory results were insignificant and shown in **Table 1**. Pulmonary function suggested partial reversible mild obstructive ventilation dysfunction and normal diffusion function, which supported the diagnosis of asthma. Further, bronchoscopy revealed several nodular protrusions along the trachea; Biopsy demonstrated lymphoid hyperplasia, with immunohistochemical examination showing lymphoma cells were positive for CD20, PAX-5, Bcl-2, CD21 (FDC net), CD23 (FDC net), CK (epithelium), Bcl-6 (partial), CD5 (T cell), Ki67 (+15%), but negative for CD10, CD30, Cyclin D1 and SOX11 (**Figure 3**). The pathogen detection in the BALF was all negative. Finally, we made a diagnosis of MALT lymphoma. After discharge, the patient was admitted to the hematology department for further evaluation. The bone marrow biopsy and gastrointestinal endoscopy indicated no lymphoma involvement. PET-CT detected bilateral lung nodules without FDG avidity.

## 3 Intervention, follow-up and outcomes

Taking into account the patient’s preference, case 1 opted for no special therapeutic intervention for MALT lymphoma and close observation. During the 9-month follow-up after pneumonia treatment, the patient showed no clinical symptoms, and the re-examination of chest CT indicated that the pneumonia had totally absorbed and the MALT lymphoma remained progression-free. Case 2 also received watchful waiting therapy due to she was asymptomatic and her MALT lymphoma was confined to the trachea. Case 2 remained stable at 7-month follow-up.

## 4 Discussion

The diagnosis of pulmonary MALT lymphoma relies on histological examination. Morphologically, it is characterized by small lymphoid cells resembling centrocyte-like cells or monocytoid cells, along with lymphoepithelial lesions and follicular colonization (8). Immunohistochemical analysis aids in differential diagnosis and confirmation. In our cases, CD20 (+), CD19 (+), and PAX-5 (+) confirmed B-cell lineage, while a Ki67 proliferation index of 15%-20% indicated its indolent nature. CD21 (FDC net+), CD23 (FDC net+) and CK (+) suggested the lymphoma invading follicular structures and the bronchial epithelium. The absence of CD10, CD30, SOX11 and Cyclin D1 help rule out other B-cell lymphoma. Additionally, PET-CT, bone marrow biopsy, gastrointestinal endoscopy, and 7-9 months of follow-up revealed no extrapulmonary involvement, infections, or other malignancies. Therefore, both cases were accurately diagnosed as primary pulmonary MALT lymphoma.

The clinical and radiological presentation of primary pulmonary MALT lymphoma is highly variable. When parenchymal involvement occurs, typical manifestations include nodules, consolidations, irregular airway wall thickening, luminal stenosis, and/or atelectasis. Approximately 70% of cases show bilateral or multifocal lesions, while 30% exhibit hilar or mediastinal lymphadenopathy (9, 10). Besides endobronchial MALT lymphoma, differential diagnosiss for endobronchial nodular lesions should also include primary and metastatic airway malignancies, benign tumors (e.g., endobronchial chondromas), infectious processes (e.g., tuberculosis), inflammatory conditions (e.g., sarcoidosis), other rare disorders (e.g., tracheobronchial ossification). It’s worth noting that Dieulafoy’s lesions can also be found in the submucosa of the bronchus, improper transbronchial biopsy may lead to massive bleeding (11). Accurate diagnosis requires comprehensive evaluation incorporating clinical presentation, imaging findings, and pathological examination.

The diagnostic efficacy of bronchoscopy is higher when it targets visible endobronchial lesions or radiographically apparent abnormalities. Yoon et al. classified CT findings of endobronchial MALT lymphoma into three patterns: isolated intraluminal nodules, multiple nodular protrusions, and diffuse wall thickening (12). Notably, 79.6% of lesions localized to central airways, with multiple nodules (60.4%) being most common, while solitary nodules and diffuse wall thickening accounted for 25% and 10.4%, respectively (13). Therefore, careful observation of trachea under bronchoscopy is crucial for diagnosing endobronchial MALT lymphoma (14). In 2018, Kawaguchi et al. reviewed 20 cases of endobronchial MALT lymphomaand highlighted that several widely stalked nodular protrusions covered with smooth bronchial mucosa were a characteristic bronchoscopic finding of endobronchial MALT lymphoma (15). Our two cases exhibited similar features: multiple widely stalked and smooth submucosal nodules or protrusions, mainly located at the central airways (trachea or bronchus). PET-CT is not routinely recommended for MALT lymphoma due to absent FDG avidity in up to 50% of cases. Low FDG uptake may be associated with small tumor size and low Ki-67 index, with typical SUVmax values ranging from 2.2 to 6.3 (16, 17), reflecting the indolent nature of MALT lymphoma.

There is no standard treatment for primary pulmonary MALT lymphoma. Options include observation, radiotherapy, immunotherapy, chemotherapy, or surgery, with excellent 5-year survival rates (87.5%–93.6%) (18). Given its indolent nature, asymptomatic or early-stage patients may benefit from watchful waiting or localized radiotherapy (19, 20). Kunye Kwak et al. reported 10-year event-free (EFS) and overall survival (OS) rates of 78.7% and 100% for localized therapies, outperforming systemic chemotherapy, which was 56.9% and 71.7%, respectively (21). Similarly, Wei Yan et al. found no OS difference between observation and rituximab-based therapy in early-stage disease (22). Besides, surgical resection may benefit patients with high tumor burden, *in-situ* progression or suspected malignant transformation. A study of 123 patients with primary pulmonary MALT lymphoma compared three first-line treatments. Results showed that OS was high in all groups (93% at 6 years), though surgery had the best results (100%), followed by active monitoring (91%) and systemic therapy (76%). Complete surgical resection had better long-term disease control (6-year EFS: 74% vs. 65% vs. 62%; *P*=0.013) (23). For inoperable patients, anti-CD20 monoclonal antibody with or without chemotherapy was effective, providing a 50% 5-year EFS and 90% OS, regardless of disease location (24).

Our study has several limitations. First, the small sample size of only two cases limits the generalizability of the findings. With low incidence, larger and multicenter studies are needed to better characterize the bronchoscopic features of primary endobronchial MALT lymphoma. Second, the follow-up period (7-9 months) was relatively short, which may not fully capture the long-term progression or outcomes of the “watch and wait” approach in such patients. Future research should focus on advanced molecular techniques assisting the early and noninvasive diagnosis. Clinical trials comparing conservative observation with localized therapies (e.g., radiotherapy) or systemic treatments (e.g., rituximab-based regimens) are also needed to establish standardized guidelines for asymptomatic or early-stage patients.

## 5 Conclusion

Primary endobronchial MALT lymphoma with isolated trachea and bronchus involvement is rare and easily misdiagnosed. Thorough bronchoscopy examination of the central airways and appropriate biopsy of characteristic lesions are important. Endobronchial MALT lymphoma should be considered when targeting multiple widely stalked and smooth submucosal nodules or protrusions, mainly located at the central airways.

## 6 Patient perspective

Because of both patients were asymptomatic and remained stable, they refused re-examination of bronchoscopy. But both patients have agreed to undergo long-term outpatient follow-up.

## Data Availability

The original contributions presented in the study are included in the article/supplementary material. Further inquiries can be directed to the corresponding author.
